# Curry Leaf Triggers Cell Death of *P. gingivalis* with Membrane Blebbing

**DOI:** 10.3390/pathogens10101286

**Published:** 2021-10-06

**Authors:** Ryoma Nakao, Tsuyoshi Ikeda, Soichi Furukawa, Yasushi Morinaga

**Affiliations:** 1 Department of Bacteriology I, National Institute of Infectious Diseases, Tokyo 162-8640, Japan; 2 Department of Pharmaceutical Sciences, Sojo University, Kumamoto 860-0082, Japan; tikeda@ph.sojo-u.ac.jp; 3 Department of Food Bioscience and Biotechnology, College of Bioresource Science, Nihon University, Kanagawa 252-0880, Japan; yasushi.morinaga@gmail.com

**Keywords:** bacteria, periodontal disease/periodontitis, antimicrobials/antimicrobial resistance, herbal medicine, curry leaf, *Porphyromonas gingivalis*, high-speed atomic force microscopy (HS-AFM), membrane blebbing, membrane depolarization

## Abstract

Periodontal disease has become a serious public health problem, as indicated by accumulating evidence that periodontal disease is not only a major cause of tooth loss but is also associated with various systemic diseases. The present study assessed the anti-bacterial activities of three herbal products (curry leaf, clove, and cinnamon) against *Porphyomonas gingivalis*, a keystone pathogen for periodontal diseases. The curry leaf extract (CLE) showed the strongest growth inhibitory activity among them, and the activity was maintained even after extensive heat treatment. Of note, while clove and cinnamon extracts at sub-minimum inhibitory concentrations (sub-MICs) significantly enhanced the biofilm formation of *P. gingivalis*, CLE at sub-MIC did not have any effect on the biofilm formation. The MIC of CLE against *P. gingivalis* was higher than those against a wide range of other oral bacterial species. *P. gingivalis* cells were completely killed within 30 min after treatment with CLE. Spatiotemporal analysis using high-speed atomic force microscopy revealed that CLE immediately triggered aberrant membrane vesicle formation on the bacterial surface. Bacterial membrane potential assay revealed that CLE induced depolarization of the bacterial membrane. Taken together, these findings suggest the mechanism behind early bactericidal activity of CLE and its therapeutic applicability in patients with periodontal diseases.

## 1. Introduction

Periodontal disease is not only a major cause of tooth loss but is also associated with a range of systemic diseases [1]. *Porphyromonas gingivalis* is a major periodontopathic bacterium that possesses a diverse repertoire of virulence factors [2] and causes disruption of host–microbial homeostasis, leading to inflammatory bone loss [3,4]. *P. gingivalis* has thus been regarded as a primary target for prevention and treatment of periodontal diseases. However, sufficient progress has not been made to develop novel drugs for periodontal diseases, such as *P. gingivalis*-selective antimicrobials, for several decades. In addition, antimicrobial resistance (AMR) is an increasingly serious threat to global public health. In response to the serious situation, the world health organization (WHO) described the importance of natural sources of biodiversity and biorepositories as sources for the development of new antibiotics to combat AMR [5].

Bioactive natural product-based medicine for the prevention or cure of periodontitis has garnered much attention from both the public and scientific communities. In addition, the therapy using natural products is generally less expensive as compared with highly pure chemicals. Moreover, it is likely that a range of natural product-based therapies could become an alternative treatment option for periodontitis [6,7]. Curry leaf (*Murraya koenigii*) is an herbal product frequently used especially in southern India for flavoring curries and chutneys. It has been reported that curry leaf has antibacterial activity against a range of pathogens, such as mycobacteria [8], oral streptococci, *Pseudomonas aeruginosa*, *Candida albicans* [9], and Plasmodium [10]. However, the anti-bacterial effect of curry leaf on *P. gingivalis* has not been investigated.

In the present study, we aimed to better understand the anti-bacterial activity of curry leaf extract (CLE) by focusing on electrophysiological/spatiotemporal features of the bacterial membrane. We found that CLE rapidly triggers cell death of *P. gingivalis* with membrane blebbing. CLE also showed a strong ionophoric activity against bacterial membrane. Based on these findings, we discuss the clinical applicability of potential new CLE-based treatments against periodontitis.

## 2. Results

### 2.1. Curry Leaf Extract (CLE) Shows Anti-Bacterial Activity Together with Thermo-Stability

**Figure 1:**
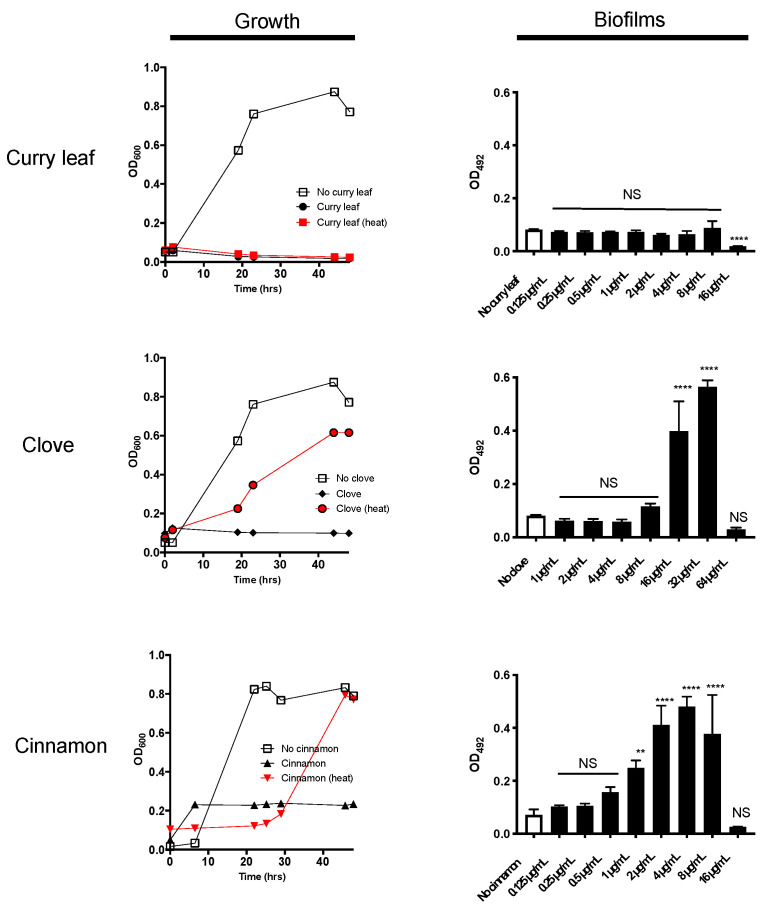
Anti-bacterial and anti-biofilm effects of three herbal products on *P. gingivalis*. (**Left panels**) Shown are growth curves of *P. gingivalis* cultured in the presence of three herbal extracts at the MICs. The MICs of curry leaf, clove, and cinnamon were 16, 64, and 16 µg/mL, respectively (data not shown). The turbidity (OD_600_) of the bacterial culture was monitored for two days at different time points. The thermo-stability of each herbal product at MICs was also assessed (denoted as points/lines of red). Data shown are representative of three independent experiments performed in triplicate assays. Similar results were obtained in three independent experiments. (**Right panels**) The effects of herbal extracts on biofilm formation of *P. gingivalis* were examined in 96-well microplate assay. The Y-axes indicate the biofilm mass after 48-h culture (OD_492_). Data shown are the mean with SD from three independent experiments performed in triplicate assays. White bars indicate the vehicle control without herbal products. Black bars indicate the herbal products at different concentrations. NS: no statistical significance. ** *p* ≤ 0.01, **** *p* ≤ 0.0001 (vs. vehicle control without herbal product).

For screening of an herbal product that shows a potential inhibitory activity against *P. gingivalis*, we chose a high-throughput assay system using 96-well plates, by which both growth and biofilm formation can be assessed at the same time. Among 12 different ethanol-extracted herbs tested, we found that curry leaf, clove, and cinnamon showed *P. gingivalis* growth inhibitory activity. The MICs of extracts of curry leaf (CLE), clove, and cinnamon was determined as 16, 64, and 16 µg/mL, respectively (Figure 1), and each extract inhibited the growth in a dose-dependent manner (data not shown). Both clove and cinnamon extracts treated at 100 °C for 15 min partially lost their growth inhibitory activities; however, CLE after heat treatment completely inhibit *P. gingivalis* growth without any loss of activity (Figure 1), indicating that CLE contains thermostable antimicrobials. In the biofilm formation assay, CLE at a concentration of 16 µg/mL significantly inhibited biofilm formation (Figure 1). Notably, biofilm formation significantly increased by the addition of extracts of clove or cinnamon at the sub-minimum inhibitory concentrations (sub-MICs). On the basis of these screening results, we decided to focus on the anti-bacterial effect of CLE in the following experiments. As a part of the authentication of CLE used in this study, the major components have been confirmed by HPLC analysis (Supplemental Figure S1) in reference to a published work characterizing curry leaf compounds [11].

### 2.2. P. gingivalis Is Highly Susceptible to Treatment with CLE

Next, we determined the MICs of three strains of *P. gingivalis*, as well as another 15 oral bacterial strains including two strains of *Fusobacterium nucleatum*, two *Prevotella* species, two strains of *Aggregatibacter actinomycetemcomitans*, and nine different species of oral streptococci. The MICs of CLE against these species are listed in Table 1. The MICs of *P. gingivalis* were 16 or 32 µg/mL, which were the lowest concentrations among the tested bacteria. Although *A. actinomycetmcomitans* ATCC 29522, *S. gordonii* ATCC 10558, *S. oralis* No. 10, and *S. sobrinus* ATCC 6715 were more resistant to CLE (64 µg/mL) than *P. gingivalis*, all tested 18 oral strains except the three species were even more resistant to CLE (more than 64 µg/mL) than *P. gingivalis*, demonstrating selective anti-bacterial activity of CLE against *P. gingivalis*. We also examined whether CLE showed toxicity on human oral epithelial cells. Treatment of the cells with CLE at the MIC against *P. gingivalis* (16 or 32 µg/mL) induced neither morphological changes nor LDH release even after 6 h (data not shown). Together, CLE at a concentration of 16 or 32 µg/mL shows selective anti-*P. gingivalis* activity without any in vitro toxicity to human cells.

### 2.3. CLE Showed Strong and Rapid Bactericidal Activity

**Figure 2:**
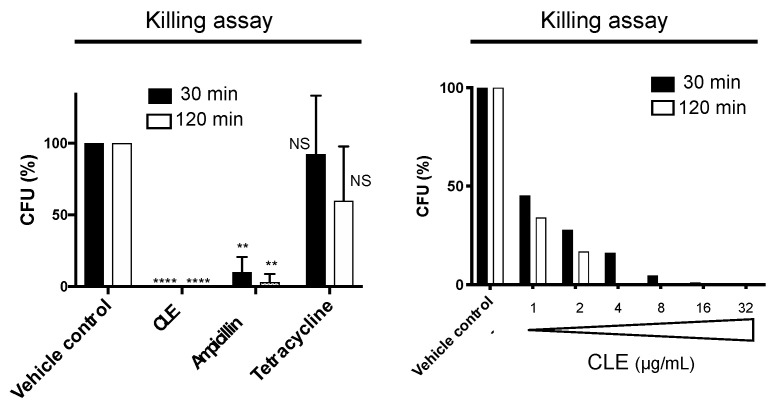
Killing activity of curry leaf extract. (Left panel) *P. gingivalis* cells were treated with curry leaf extract (CLE) at MIC (16 μg/mL) or the vehicle control (ethanol) for 30 or 120 min. Ampicillin and tetracycline were also used at concentrations of 100 μg/mL (ca. 6000-fold MIC) and 5 μg/mL (not less than 100-fold MIC), respectively. Viability of *P. gingivalis* was evaluated by counting CFUs on BAPs after culture for 14 days. The survival rate is shown as (CFU [tested sample]/CFU [vehicle control]) × 100 (%) at each time point. Data are expressed as the mean with SD from results obtained in three independent experiments. NS: no statistical significance. ** *p* ≤ 0.01, **** *p* ≤ 0.0001 (vs. vehicle control). (Right panel) *P. gingivalis* cells were treated with CLE at the concentrations ranging from 1 to 32 µg/mL, or the vehicle control (1% ethanol) for 30 and 120 min. Shown are the survival rates after treatment, compared to the vehicle control. The rate denoted as CFU (%) was calculated as (CFU [tested sample]/CFU [vehicle control]) × 100 (%).

To better understand the anti-bacterial activity of CLE, we performed a killing assay by counting CFUs of *P. gingivalis* after treatment with CLE at the MIC (Figure 2). Two standard antibiotics, ampicillin and tetracycline, that show bactericidal and bacteriostatic effects, respectively, were also used as controls (Figure 2). Treatment with tetracycline for 30 or 120 min did not significantly reduce the CFUs as compared to the vehicle control (Figure 2). Treatment with ampicillin for 30 or 120 min dramatically reduced the CFUs to approximately 10% or 5% of that of the vehicle control, respectively (Figure 2). Surprisingly, complete killing was achieved within 30 min after adding CLE (Figure 2). This killing activity occurred in a dose-dependent manner (Figure 2). These results demonstrated that CLE showed strong bactericidal effects on *P. gingivalis*, and the bactericidal action of CLE occurred more rapidly than that of ampicillin, a standard β-lactam with bactericidal activity.

### 2.4. CLE Attacks Bacterial Outer Membrane

**Figure 3:**
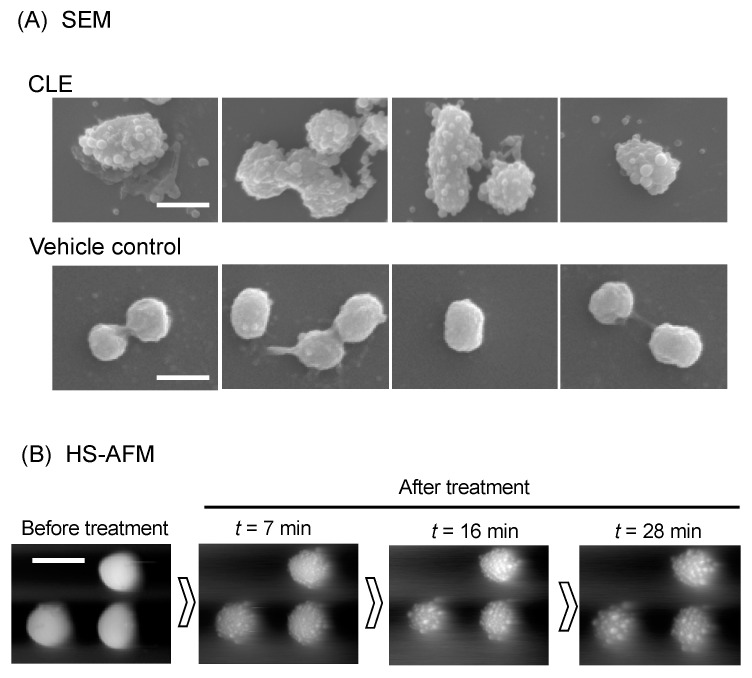
Morphological change of *P. gingivalis* cells. (**A**) SEM analysis. *P. gingivalis* cells were treated with CLE at MIC or vehicle control for 30 min. Four different areas of CLE- and vehicle control-treated cells were shown. Scale: 1000 nm. (**B**) High-speed atomic force microscopy (HS-AFM) analysis. *P. gingivalis* cell morphology was monitored with a BIXAM system (Olympus) at nanometer scale. *P. gingivalis* cells were immobilized on glass slides and treated with CLE at 4-fold MIC (64 µg/mL). Nanometer-scale dynamics were continuously monitored. Shown are images of the cells before (Pre) and 7, 16, and 28 min after treatment with curry leaf (*t* = 7, 16, and 28). See also Supplemental Movies S1 and S2.

SEM analysis revealed that treatment with CLE dramatically altered the appearance of outer membrane of *P. gingivalis* (Figure 3). The whole cell surfaces were covered by numerous, aberrant blebs, and the blebs were also released into the extracellular milieu (Figure 3). We also examined the nanometer-scale dynamics of CLE-treated *P. gingivalis* cells using a high-speed atomic force microscopy (HS-AFM) system as an alternative to nanometer-scale imaging. Spatiotemporal HS-AFM analysis showed that CLE triggered numerous membrane blebbing at least at 7 min and the number and size of the blebs continuously increased over time for 28 min (Figure 3, and Supplemental Movies S1 and S2).

**Figure 4:**
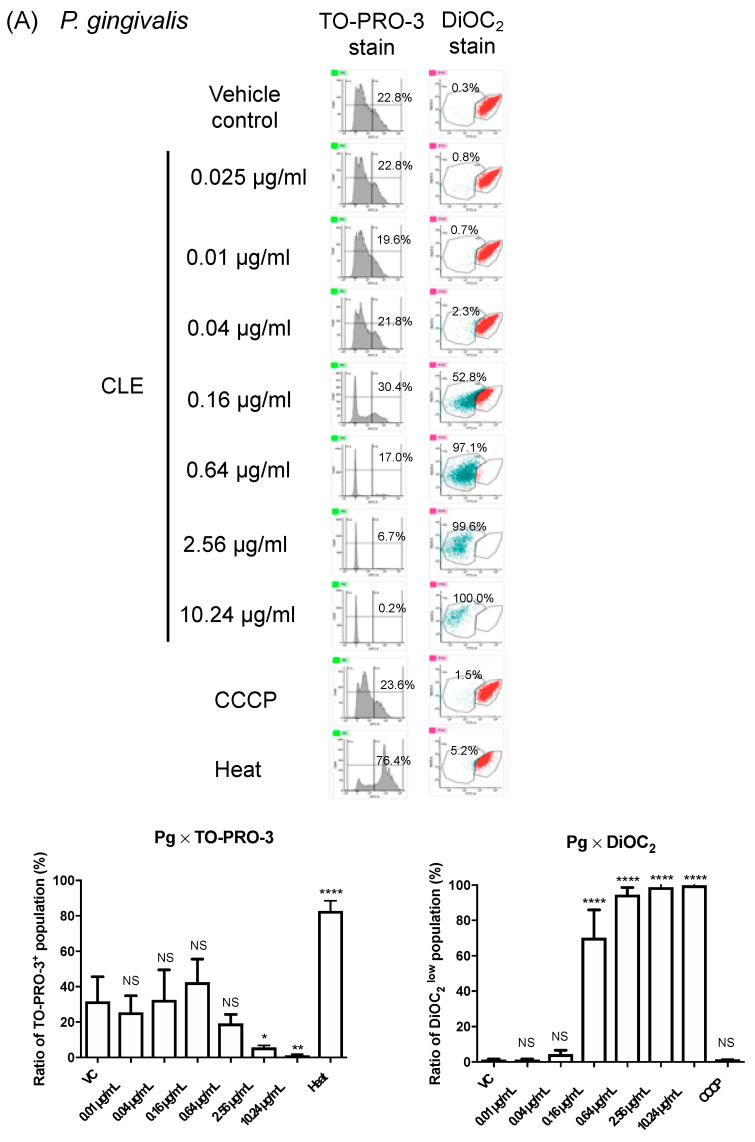

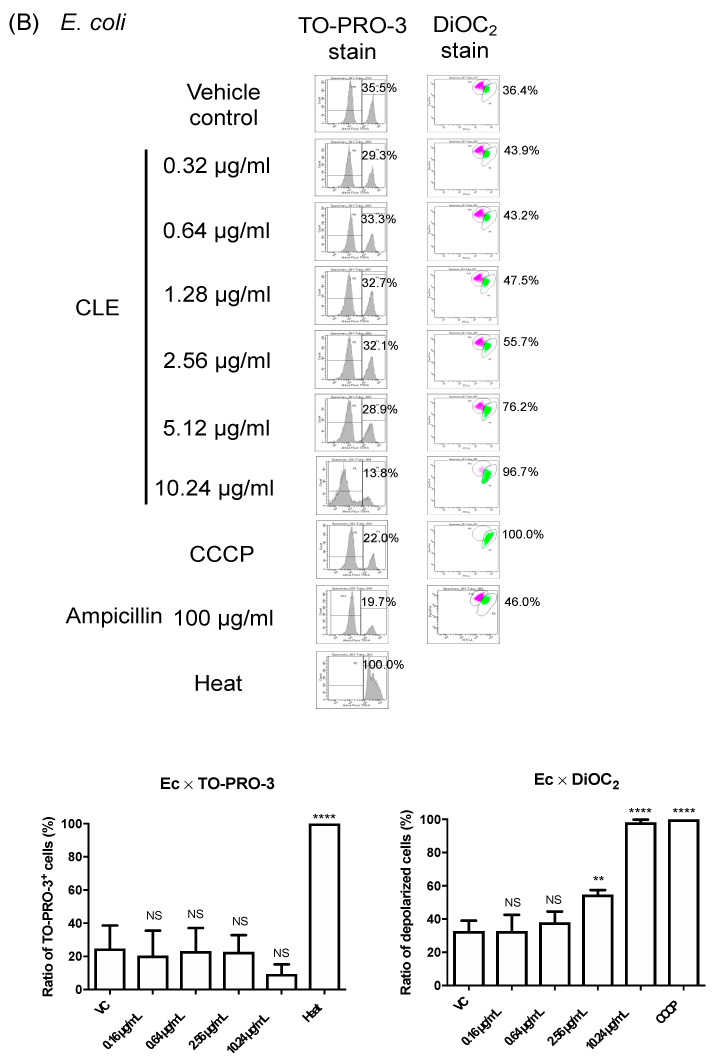
Electrophysiological analysis of bacterial membranes treated with CLE. For FACS analysis, cells of *P. gingivalis* (**A**) and *E. coli* (**B**) were treated with CLE at different concentrations as indicated, then their membrane physiology was assessed using two different fluorescent dyes, TO-PRO-3 and DiOC_2_(3). *P. gingivalis* (**A**) and *E. coli* (**B**) cells were also subjected to heat treatment (Heat) or CCCP treatment (CCCP) as controls of cells with increased membrane permeability and depolarized cells, respectively. In (**B**), another sample was also exposed to ampicillin (as a representative of β-lactams). A representative FACS data are shown in the left panels of (**A**,**B**). In the upper right bar graphs of (**A**,**B**), the y-axis shows percentages of cells with increased membrane permeability (TO-PRO-3^+^) to total cells of *P. gingivalis* (**A**) and *E. coli* (**B**). In the lower right bar graphs of (**A**,**B**), the *y*-axis shows percentages of cells showing low fluorescent intensity of DiOC_2_ (DiOC_2_^low^) in *P. gingivalis* (**A**), and percentages of cells with membrane depolarization in *E. coli* (**B**). Data of the bar graphs are shown as the mean with SD of results obtained in three or four independent experiments in (**A**) or (**B**), respectively. NS: no statistical significance. * *p* ≤ 0.05, ** *p* ≤ 0.01, **** *p* ≤ 0.0001 (vs. vehicle control).

To further characterize the influence of CLE on *P. gingivalis*, we examined how CLE affects the membrane physiology. Using *P. gingivalis* strain ATCC 33277 and a membrane impermeable dye TO-PRO-3, we firstly monitored membrane permeability to CLE (Figure 4A). Surprisingly, sublethal and lethal doses of CLE significantly decreased the membrane permeability (Figure 4A). We also examined the membrane potential of *P. gingivalis* using DiOC_2_(3)-based assay [12]. We found that the fluorescence intensity of this membrane potential-sensitive dye, significantly, was dramatically decreased by treatment of CLE in a dose-dependent manner (Figure 4A), assuming that CLE affected the membrane potential. However, we could not assert that CLE affected the membrane potential of *P. gingivalis* because no change in the signal intensity of DiOC_2_(3) was observed, even when *P. gingivalis* cells were treated with an ionophoric compound, CCCP (Figure 4A). Thus, the DiOC_2_(3)-based membrane potential assay with DiOC2(3) was not functional with *P. gingivalis*. Nevertheless, these findings suggest that the killing activity of CLE against *P. gingivalis* may not be due to the loss of membrane integrity but rather to the abnormality of the bacterial membrane accompanied with disorder of the membrane permeability and membrane potential. To determine whether CLE certainly has ionophoric activity, we performed the same assays using *E. coli* strain BW25113 (Figure 4B), which was highly resistant to CLE (MIC > 64 μg/mL, Table 1). The *E. coli*’s natural resistant property to CLE could offer a methodological advantage because bacterial membrane response after treatment with CLE might be investigated in a broad concentration range without inducing cell death. The results of the assays revealed that CLE induced membrane depolarization in a dose-dependent manner without increasing membrane permeability (Figure 4B). Notably, CLE at 10.24 μg/mL induced membrane depolarization nearly as strongly as a representative ionophore, CCCP. On the other hand, the ionophoric activity in ampicillin was not observed (Figure 4B).

## 3. Discussion

*P. gingivalis* is regarded as a late colonizer of biofilms in subgingival pockets, as well as a pathobiont that leads to dysbiosis in oral cavity [13]. In the present study, we found that biofilm formation of *P. gingivalis* was strongly enhanced by sub-MICs of ethanol extracts of clove and cinnamon (Figure 1). Some antibiotics or disinfectants at sub-MIC have been reported to induce biofilm formation of some bacterial pathogens, such as *Staphylococcus aureus* [14], *Pseudomonas aeruginosa* [15], *Staphylococcus epidermidis* [16], *Staphylococcus* *saprophyticus*, and uropathogenic *Escherichia coli* [17]. To the best of our knowledge, this is the first report of *P. gingivalis* biofilm formation enhanced by low doses of natural products with anti-bacterial activities. Therefore, we would like to call attention to the possibility that biofilm formation is enhanced by low doses of anti-*P. gingivalis* products such as clove and cinnamon, such that the increased biofilm formation might deteriorate the subgingival environment and develop periodontal diseases.

The basis for every living organism depends on the ability of the cell to maintain ion gradients across biological membranes, i.e., membrane potential. Ionophoric drugs are attractive in terms of its application to a wide range of pathogens. In general, ionophoric antibiotics induce cell death through creating an imbalance in the cytoplasmic membrane’s ion leak-pump relationship. For example, valinomycin is a representative ionophoric antibiotic with K^+^-selective ionophoric activity, resulting in an increase in K^+^ permeability of the membrane. Gramicidin, a representative peptide ionophore, forms a continuous channel that spans the membrane. Very recently, Hards et al. reported a novel ionophoric antibiotic candidate that can inhibit ATP synthesis in *E. coli* by functioning as a H^+^/K^+^ ionophore, causing transmembrane pH and potassium gradients to be equilibrated [18]. In terms of the difference in sensitivity of between Gram-positive and Gram-negative bacteria to ionophores, Tempelaars et al. reported that both cereulide and valinomycin, two antibiotics that share highly similar cyclic dodecadepsipeptides with K^+^-selective ionophoric activity, showed anti-bacterial activity against Gram-positive bacteria, but not against Gram-negative bacteria [19]. The difference in sensitivity between Gram-negative and Gram-positive bacteria may be due to the inability of these ionophores to cross the outer membrane. On the other hand, in the present study, FACS analysis using an *E. coli* membrane model revealed that CLE showed striking ionophoric activity against the cytoplasmic membrane of even Gram-negative bacteria. The activity of CLE at 10-fold MIC (against *P. gingivalis*) was nearly as strong as that of CCCP at the final concentrations of 10 µM, which is known to induce cytoplasmic membrane depolarization of Gram-positive bacteria as well as Gram-negative bacteria (Figure 4). In addition, notably, *P. gingivalis*, a Gram-negative anaerobe, was the most sensitive species among the oral bacterial species examined in this study, while Gram-positive oral commensals were relatively insensitive. These findings suggest the applicability of CLE as a narrow-spectrum therapeutic to treatment of periodontitis, if CLE would selectively eliminate *P. gingivalis* in the periodontal pockets while maintaining the homeostatic benefit provided by oral commensals.

In a previous report regarding chemical composition analysis, curry leaf extract contains plentiful and varied alkaloids and flavonoids [11], which is in agreement with the present study showing the presence of a range of alkaloids in CLE by HPLC analysis with the leading compound mahanine (Supplemental Figure S1). Mahanine, a novel carbazole alkaloid derived from curry leaf, inhibits the growth of prostate cancer cells via blocking androgen receptor signaling [20] and also induces cell death in pancreatic adenocarcinoma cells by inducing reactive oxygen species production [21], thereby implicating a therapeutic role for mahanine in cancer treatment. On the other hand, no mechanistic insight into the antibacterial effect of curry leaf in infectious disease research has been shown. Regarding the antimicrobial compounds, further biochemical and functional investigations should proceed on the basis of the chromatographical analysis of CLE (Supplemental Figure S1). Nevertheless, we suggest that curry leaf contains compound(s) with strong ionophoric activity that are responsible for its anti-bacterial activity to both Gram-positive and Gram-negative bacteria.

It has been also reported that curry leaf showed anti-inflammatory effect in in vitro [22] and clinical studies [23]. Adebajo et al. reported that Methanol-extracted curry leaf shows antioxidant and anti-inflammatory properties and proposed applicability for the treatment of gastro-intestinal inflammation, bronchitis, and hepatitis [24]. Therefore, CLE may also be useful for prevention of periodontal diseases, in an alternative view of attenuation of gingival inflammation. Further studies to clarify the possible therapeutic effect on inflamed gingival tissues in patients are needed.

Antimicrobial resistance (AMR) of bacterial pathogen is a serious risk to global health. The WHO’s “global action plan on AMR” [5] was followed by worldwide support from governments, health ministries, and health agencies. However, despite such extensive efforts, researchers are still in the process of establishing scientific and rational strategies to combat globally emerging and re-emerging infectious diseases. In particular, regarding development of antibiotics, no new class of antibiotics with activity against Gram-negative bacteria has been approved in over fifty years. In the context of natural products that combat pathogens, we have provided here a feasible strategy to selectively kill *P. gingivalis*. Future studies to identify the responsible compound in curry leaf and to better understand the mechanisms behind its activity would further propose the therapeutical applicability of curry leaf for treatment of other infectious diseases.

## 4. Materials and Methods

### 4.1. Herbs and Preparation of Ethanol-Extracted Herb

Dried whole curry leaf, clove powder, and cinnamon powder were purchased from S&B Food Inc. (Tokyo, Japan), which is approved by international organization for standardization (ISO) 9001. The dried whole curry leaves were ground into a fine powder by using a hand mill CM-50GT (Kyocera Corp., Kyoto, Japan). Curry leaf (*Murraya koenigii*) used in this study was collected in the north western province of Sri Lanka, situated between 7°28′04″ and 8°55′20″ N latitude and between 79°68′58″ and 80°56′90″ E longitude in 2015. Clove (*Syzygium aromaticum*) was collected in Indonesia, situated between 5°38′90″ N and 10°99′25″ S latitude and between 95°25′64″ and 140°78′38″ E longitude in 2015. Cinnamon (*Cinnamomum cassia*) was collected in the Guangxi Zhuang autonomous region of China, situated between 21°40′06″ and 26°37′23″ N latitude and between 104°45′94″ and 112°07′49″ E longitude in 2015. To prepare each herbal extract, 100 mg of the powder was stirred in 2 mL of 100% ethanol for 15 min at room temperature. After stirring, the extract in the supernatant after centrifugation was collected, and the concentration of each extract was determined as its protein content based on Bradford assay [25]. The extract was concentrated after evaporation by centrifugation under vacuum and was eventually redissolved with ethanol to standardize at 6.4 mg/mL. Thus, ethanol was used as solvent for all herbal extracts in our experiments. Regarding curry leaf extract used in this study, the chromatographic analysis has been also performed (Supplemental Figure S1).

### 4.2. Bacterial Strains and Growth Conditions

All strains used in this study are shown in Table 1. All *Porphyromonas* and *Prevotella* strains were grown in brain heart infusion (BHI) broth (Beckton Dickinson Co., Franklin Lakes, NJ, USA) supplemented with hemin (5 µg/mL) (Fujifilm Wako Chemicals Co., Tokyo, Japan) and menadione (1 µg/mL) (Fujifilm Wako Pure Chemicals, Osaka, Japan), or on BHI blood agar plates (BAP) containing hemin and menadione. *Porphyromonas gingivalis* strain ATCC 33277 was mainly used for this study to screen the herbal products for anti-bacterial activity as well as to further examine the activity in detail. BHI broth and BHI BAP (without hemin and menadione) were used to maintain strains of the other oral bacteria including streptococci and *Aggregatibacter actinomycetmcomitans*, *Fusobacterium nucleatum*. All oral bacteria were grown in an anaerobic chamber (miniMACS anaerobic workstation; Don Whitley Scientific Ltd., Shipley, UK) in 80% N_2_, 10% H_2_, and 10% CO_2_ at 37 °C. A laboratory *Escherichia coli* strain BW25113, which was used for bacterial membrane potential assay, is maintained in LB (Becton Dickinson) broth and on LB agar under aerobic conditions.

### 4.3. Growth Assay and Biofilm Formation Assay

Strain ATCC 33277 was used for the growth and biofilm formation assay of *P. gingivalis*. Growth in the presence of the herbal extracts at different concentrations was monitored at different time points, using 96-well plates (3595; Corning, New York, NY, USA). Two-fold dilution series of each herbal extracts of curry leaf, clove, and cinnamon (2.5 µL) were added to the bacterial suspension (200 µL, 1 × 10^8^ CFU/mL) in BHI-HM broth. The turbidity of the bacterial suspension (OD_620_) during growth was measured at different time points using a micro-plate reader (Cytation 5, BioTek, Winooski, VT, USA). Bacterial strains listed in Table 1 were assayed for determination of MICs. The MIC for each strain was determined using microdilution methods according to the protocols of the Clinical and Laboratory Standards Institute [26,27], with some modifications. MIC was defined as the minimum concentration that restricted growth to a level of A_600_ < 0.05 at after incubation for 48 h. The broth contained a 2-fold dilution series of extract of each herbal product at final concentrations ranging from 0.125 to 64 µg/mL. After monitoring the growth for two days, biofilm formation was also assessed using the same 96-well plates, as described previously [28]. In brief, 2-day-old biofilms grown in the 96-well plates under static conditions were stained with 0.25% safranin. All dye associated with the attached biofilms was dissolved with 80% ethanol, and then the optical density at 492 nm (OD_492_) was measured using a micro-plate reader (Cytation 5, BioTek, Tokyo, Japan).

### 4.4. Killing Assay to Assess Bactericidal Activity

The bactericidal activity of the curry leaf extract against *P. gingivalis* was evaluated at 30 and 120 min using a killing assay, as described previously in our laboratory [29].

### 4.5. Scanning Electron Microscopy (SEM)

SEM analysis of *P. gingivalis* cells treated with the curry leaf extract for 30 min was performed using an S-5200, (HITACHI, Hitachi, Japan), as described previously in our laboratory [30].

### 4.6. Spatiotemporal Analysis Using High-Speed Atomic Force Microscopy (HS-AFM)

Spatiotemporal analysis using HS-AFM was performed using the BIXAM real-time imaging system (Olympus Corp., Tokyo, Japan), as described previously [29,31,32,33]. Prior to HS-AFM, *P. gingivalis* cells were labeled with the fluorescence dye rhodamine (excitation/emission of 543/580 nm) using NHS-rhodamine (Thermo Fisher Scientific, Waltham, MA, USA) according to the manufacturer’s instructions. For immobilization, rhodamine-labeled cells were incubated on glass slides (SF17370, Matsunami glass, Osaka, Japan) for 5 min. The commercially available cantilever BL-AC10DS-A2 (Olympus Corp.) and USC-F0.8-k0.1 (Nanoworld AG, Neuchâtel, Switzerland) was used for analysis.

### 4.7. Cytotoxicity toward Oral Epithelial Cells

Cytotoxicity of the samples toward the oral squamous cell carcinoma cell line Ca9.22 was evaluated by quantifying release of lactate dehydrogenase (LDH) with plasma membrane damage (Cytotoxicity Detection Kit Plus (LDH), Roche Diagnostics GmbH, Mannheim, Germany), according to the manufacturer’s instructions with some modifications, as described previously in our laboratory [29].

### 4.8. Bacterial Membrane Potential Assays

Bacterial membrane potential assays using BW25113 strain were performed in the presence of a membrane potential indicator dye, 3,3′-diethyloxacarbocyanine iodide (DiOC_2_ [3], AAT Bioquest, Inc., Sunnyvale, CA, USA) and a membrane-impermeable dye TO-PRO-3, as described previously [12,29].

### 4.9. Statistical Analysis

Statistical analysis was performed using the Mann–Whitney U test or one-way analysis of variance (ANOVA), followed by Dunnett’s multiple comparison test. *p*-values of 0.05 or less were considered to be statistically significant.

## Figures and Tables

**Table 1:** MIC (μg/mL) of CLE against 18 oral bacterial strains and *E. coli*.

Strains	MICs
*Porphyromonas gingivalis* ATCC 33277	16
*Porphyromonas gingivalis* W83	16
*Porphyromonas gingivalis* W50	32
*Fusobacterium nucleatum* #20	>64
*Fusobacterium nucleatum* ATCC 23726	>64
*Prevotella loescheii* ATCC 15930	>64
*Prevotella nigrescens* ATCC 33563	>64
*Aggregatibacter actinomycetemcomitans* Y4	>64
*Aggregatibacter actinomycetemcomitans* ATCC 29522	64
*Streptococcus anginosus* ATCC 33397	>64
*Streptococcus cristatus* ATCC 51100	>64
*Streptococcus gordonii* ATCC 10558	64
*Streptococcus mitis* ATCC 6245	>64
*Streptococcus mutans* UA159	>64
*Streptococcus oralis* No. 10	64
*Streptococcus salivalius* ATCC 9759	>64
*Streptococcus sanguinis* ATCC 10556	>64
*Streptococcus sobrinus* ATCC 6715	64
*Escherichia coli* BW25113	>64

## Data Availability

Not applicable.

## Supplementary Materials

The following are available online at https://www.mdpi.com/article/10.3390/pathogens10101286/s1. Supplemental Figure S1: HPLC profile of compounds in CLE, Supplemental Movie S1: Real-time observation of the *P. gingivalis* cell surface before treatment with CLE, Supplemental Movie S2: Real-time observation of the *P. gingivalis* cell surface after treatment with CLE.
