# Supplementary material for: Curry Leaf Triggers Cell Death of P. gingivalis with Membrane Blebbing
Source: Pathogens. 2021 Oct 6;10(10):1286. doi: 10.3390/pathogens10101286 (PMC8538566; doi:10.3390/pathogens10101286)
Supplement: Supplementary file 1 [file pathogens-10-01286-s001.zip › pathogens-1380599-suppl-figure S1.pdf]

Supplemental Figure S1. HPLC profile of compounds contained in CLE

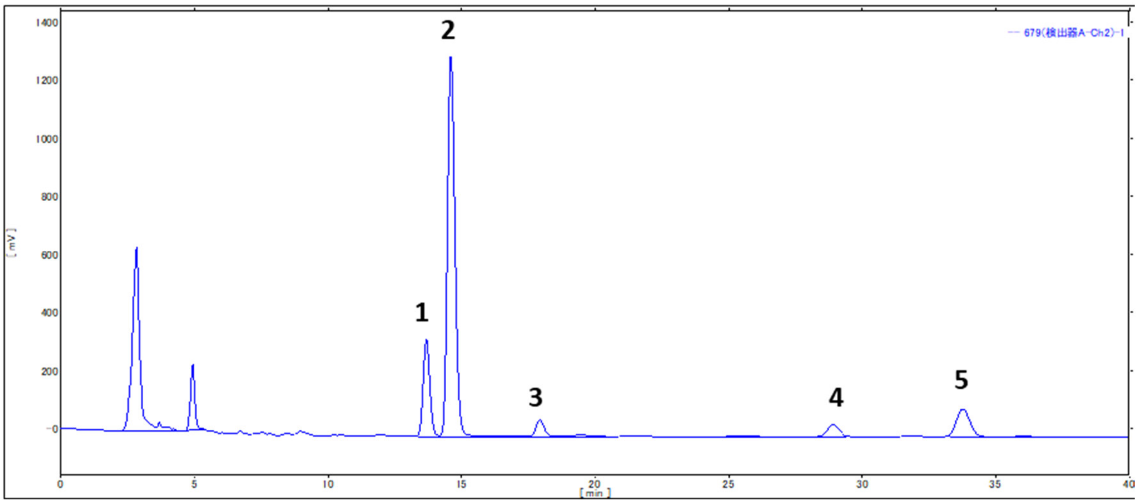

The peak number corresponds to the compound identified in the authentic sample. **1**: isomahanine, **2**: mahanine, **3**: murrayanol, **4**: mahanimbicine, **5**: mahanimbine. HPLC profiling was analyzed on a SIMADZU LC-10AD pump, SIMADZU SPD-10A detector, SIMADZU CTO-10AC column heater, and column of COSMOSIL $5C_{18}$ AR-II (5μm, ϕ4.6×250 mm, Nacalai Tasque Inc., Kyoto, Japan); Flow rate, 1.0 mL/min.; column temperature, 40 °C; Detection wavelength, 254 nm; Eluting solvent, 80% MeOH in 0.1% trifluoroacetic acid. The curry leaf extract was dissolved in EtOH at 2.56 mg/mL concentration and injected to the HPLC system (5 μL).
